# Frontline use of rituximab may prevent ADAMTS13 inhibitor boosting during caplacizumab treatment in patients with iTTP: post hoc analysis of a phase 2/3 study in Japan

**DOI:** 10.1186/s12959-024-00642-3

**Published:** 2024-08-02

**Authors:** Kazunori Imada, Yoshitaka Miyakawa, Satoshi Ichikawa, Hitoji Uchiyama, Yasunori Ueda, Yasuhiro Hashimoto, Masashi Nishimi, Masako Tsukamoto, Sayaka Tahara, Masanori Matsumoto

**Affiliations:** 1 Department of Hematology, Japanese Red Cross Osaka Hospital, Osaka, Japan; 2 Department of Hematology, Saitama Medical University Hospital, Saitama, Japan; 3 Department of Hematology, Tohoku University Hospital, Sendai, Japan; 4 Department of Hematology, Japanese Red Cross Kyoto Daiichi Hospital, Kyoto, Japan; 5 Department of Hematology Oncology, Kurashiki Central Hospital, Kurashiki, Japan; 6 Sanofi K.K, Tokyo, Japan; 7 Department of Hematology and Blood Transfusion Medicine, Nara Medical University Hospital, 840 Shijyo-Cho, Kashihara, Nara, 634-8522 Japan

**Keywords:** Caplacizumab, ADAMTS13 inhibitors, ADAMTS13 activity, Immune-mediated thrombotic thrombocytopenic purpura, TTP

## Abstract

**Background:**

A recent Phase 2/3 study in Japanese patients showed that caplacizumab was effective in treating immune-mediated thrombotic thrombocytopenic purpura (iTTP), with a low rate of iTTP recurrence. ADAMTS13 activity is monitored weekly during caplacizumab treatment to guide discontinuation of caplacizumab and consequently avoid exacerbations or relapse. The aim of this study was to assess changes in ADAMTS13 activity/inhibitor levels during caplacizumab treatment in this patient population.

**Methods:**

A post hoc analysis of the Phase 2/3 study in Japanese patients was conducted. Patients ≥ 18 years old with confirmed iTTP received 10 mg of caplacizumab daily in conjunction with therapeutic plasma exchange (TPE) and immunosuppression for 30 days post-TPE. Outcomes included time to recovery of ADAMTS13 activity, ADAMTS13 activity level at treatment end, incidence of ADAMTS13 inhibitor re-elevation (ie, inhibitor boosting) during treatment, time to platelet count recovery, number of days of TPE, and safety. Outcomes according to presence of inhibitor boosting were also assessed.

**Results:**

Nineteen patients had confirmed iTTP and were included in this analysis. Median (95% confidence interval) time to recovery of ADAMTS13 activity to ≥ 10%, ≥ 20%, and ≥ 60% was 14.6 (5.9–24.8), 18.5 (5.9–31.8), and 47.5 (18.5–60.9) days, respectively. Median (range) ADAMTS13 activity level at caplacizumab treatment end was 62.0% (29.0–101.0). Nine patients had ADAMTS13 inhibitor boosting. Delayed response of ADAMTS13 activity was observed in patients with inhibitor boosting. The median time to platelet count response and median number of TPE days were shorter in patients with inhibitor boosting compared with patients without inhibitor boosting. Rituximab was administered to almost all patients with inhibitor boosting (88.9%), after completion of TPE. Patients without inhibitor boosting who were treated with rituximab received it prior to completion of TPE. Only one patient experienced a recurrence, which occurred shortly after caplacizumab discontinuation due to an adverse event.

**Conclusions:**

In patients with iTTP, caplacizumab with TPE and immunosuppression may reduce the risk of ADAMTS13 inhibitor boosting if rituximab is administered early in the iTTP treatment period. Early administration of rituximab in addition to caplacizumab may prevent iTTP recurrence with inhibitor boosting.

**Trial registration:**

NCT04074187.

## Background

Immune-mediated thrombotic thrombocytopenic purpura (iTTP) is a rare, life-threatening disorder characterized by the formation of blood clots in small blood vessels, leading to their occlusion and consequent blood flow restriction to vital organs [1]. Possible outcomes of this occlusion include microangiopathic hemolytic anemia and severe thrombocytopenia [2]. iTTP is caused by an autoantibody-mediated severe deficiency of a disintegrin and metalloproteinase with thrombospondin type 1 motif, member 13 (ADAMTS13), leading to the accumulation of ultra-large von Willebrand Factor (VWF) multimers, which bind platelets and form microvascular thrombi [1].

Until recently, the standard treatment regimen for iTTP was therapeutic plasma exchange (TPE) and immunosuppressive therapy (corticosteroids and rituximab) [3]. Caplacizumab was developed to target the A1 domain of VWF, which prevents the binding of platelets to VWF and thus reduces the risk of microvascular thrombosis [1, 3–6]. International Society on Thrombosis and Haemostasis 2020 guidance recommends caplacizumab to be added to TPE and immunosuppressive therapy in patients with iTTP [3]. Evidence from the TITAN and HERCULES clinical trials, along with real-world studies from Germany, France, Spain, and the UK indicate that the addition of caplacizumab treatment is associated with rapid recovery of platelets [4, 7–10]. Caplacizumab was approved for use in Japan in 2022, based on the primary result of the Japan Phase 2/3 study, which demonstrated that caplacizumab was effective in Japanese patients with iTTP, with a low rate of iTTP recurrence (i.e. recurrent thrombocytopenia after initial recovery if platelet count ≥ 150 × 10^9^/L with stop of daily TPE requiring re-initiation of daily TPE) [11].

Careful monitoring of ADAMTS13 activity is required to avoid exacerbations (i.e. platelet count < 150 × 10^9^/L following a clinical response, within 30 days of stopping TPE) or relapses (i.e. platelet count < 150 × 10^9^/L following clinical remission, > 30 days after stopping TPE) due to early discontinuation of caplacizumab [4, 7, 10, 12]. Re-elevation of ADAMTS13 inhibitor levels (ie, inhibitor boosting) after completion of TPE may lead to a decrease in ADAMTS13 activity and subsequent recurrence of TTP [13]. A previous study in 52 Japanese patients with iTTP found that poor response to TPE was associated with ADAMTS13 inhibitor boosting [13]. Changes in ADAMTS13 activity/inhibitor levels are routinely captured while caplacizumab treatment is ongoing. Therefore, the aim of this post hoc analysis of the Japan Phase 2/3 study was to assess changes in ADAMTS13 activity and inhibitors over time during caplacizumab treatment in patients with iTTP.

## Methods

### Study population

This was a post hoc analysis of the Phase 2/3, prospective, single-arm, open-label study (NCT04074187) conducted at 15 centers in Japan between October 2019 and May 2021 (registration date 14th August 2019 in ClinicalTrials.gov) [11, 14]. Japanese patients (≥ 18 years old) with a clinical diagnosis of iTTP (initial or recurrent; confirmed ADAMTS13 level < 10%) who required initiation of daily TPE and had received ≤ 1 prior TPE were included. Exclusion criteria included platelet count ≥ 100,000/μL, serum creatinine level > 2.3 mg/dL (only in cases where platelet count was > 30,000/μL, so as to exclude atypical hemolytic uremic syndrome), known other causes of thrombocytopenia and known chronic treatment with anticoagulant treatment that could not be stopped.

### Study design and treatment

Details of the study design have been previously described [11]. Briefly, after confirmation of eligibility, patients received 10 mg caplacizumab once daily (first dose administered intravenously at least 15 min prior to TPE and subsequent doses administered subcutaneously after TPE) with TPE and immunosuppression for a period of variable duration. Patients received 10 mg caplacizumab once daily (subcutaneously) for ≥ 30 days post-TPE, and were then followed for 4 weeks after treatment end. Patients with persistent ADAMTS13 deficiency < 10% or other symptoms of underlying disease activity were allowed to undergo treatment extension for up to 8 weeks.

### Outcomes and assessments

Outcomes of this post hoc analysis included patient characteristics, time to platelet count recovery, time to normalization of all 3 organ damage markers (lactate dehydrogenase, cardiac troponin I, and serum creatinine), safety, time to recovery of ADAMTS13 activity, ADAMTS13 activity at end of treatment, and incidence of ADAMTS13 inhibitor boosting during treatment. Outcomes according to presence of inhibitor boosting were also assessed. The definition of very severe disease at baseline was French severity score ≥ 3, or severe neurological involvement (eg, coma, seizures, focal deficit), or cardiac involvement (ie, cardiac troponin level > 2.5 × upper limit of normal [ULN]). French severity score is defined as: Cerebral involvement: yes = 1; no = 0; Lactate dehydrogenase: > 10 × ULN = 1; ≤ 10 × ULN = 0; Age: > 60 years = 2; > 40– ≤ 60 years = 1; ≤ 40 years = 0 [15]. ADAMTS13 activity was assessed by enzyme immunoassay and ADAMTS13 inhibitors were assessed by the Bethesda assay [16]. ADAMTS13 activity and inhibitor titer levels were both evaluated on Day 1 of the daily TPE period, then weekly until the end of the study, and at the first and last follow-up visits. In this manuscript, inhibitor boosting was defined by inhibitor levels decreasing but then re-elevating (inhibitor titer ≥ 1 BU/mL), regardless of degree of decrease. The modified intention-to-treat (mITT) and safety population were defined as all patients who received at least one administration of caplacizumab.

### Statistical analyses

No formal statistical testing was conducted due to the post hoc nature of the analysis. Data are presented descriptively.

## Results

### Patient disposition and baseline characteristics

Twenty-one patients were enrolled and treated with ≥ 1 dose of caplacizumab (mITT and safety population). Overall, 19 patients had confirmed iTTP and were included in these analyses (less severe iTTP, n = 8; very severe iTTP, n = 11). Median (range) age was 59.0 (22–86) years and median (range) platelet count at baseline was 23.0 (8–78) × 10^9^/L (Table 1). The number of patients experiencing an initial TTP episode was 14 (73.7%), while 5 (26.3%) patients were experiencing a subsequent TTP episode. The median (range) of ADAMTS13 inhibitor was 1.70 (0.5–8.3) BU/mL. Seven patients had treatment extension (4 had very severe iTTP and 3 had less severe iTTP). Four patients discontinued caplacizumab treatment due to physician decision and/or adverse events, and 15 patients completed the study. At baseline, 7/19 patients had severe anemia (i.e. hemoglobin levels ≤ 80 g/L). Overall, 16/19 patients had hemoglobin data available at Weeks 4–5 after starting treatment; of these, all displayed hemoglobin values ≥ 80 g/L (range: 115–147 g/L).

**Table 1:** Patient baseline characteristics

	**Overall (*n* = 19)**
Gender, n (%)	
Male	9 (47.4)
Female	10 (52.6)
Age (years), median (range)	59.0 (22–86)
Previous TTP episode, n (%)	
No	14 (73.7)
Yes	5 (26.3)
Disease severity, n (%)	
Very severe	11 (57.9)
Less severe	8 (42.1)
Platelet count (10^9^/L), median (range)	23.0 (8–78)
Serum creatinine (µmol/L), median (range)	70.5 (49–226)
Lactate dehydrogenase (U/L), median (range)	575.0 (227–1794)
Cardiac troponin level (µg/L), median (range)	0.12 (0.03–7.47)
ADAMTS13 activity, n (%)	
< 10%	18 (94.7)
≥ 10%	1 (5.3)
ADAMTS13 inhibitor (BU/mL), median (range)	1.70 (0.5–8.3)

ADAMTS13, a disintegrin and metalloproteinase with thrombospondin type 1 motif member 13; iTTP, immune-mediated thrombotic thrombocytopenic purpura

### Changes to ADAMTS13 inhibitor levels

**Fig. 1:**
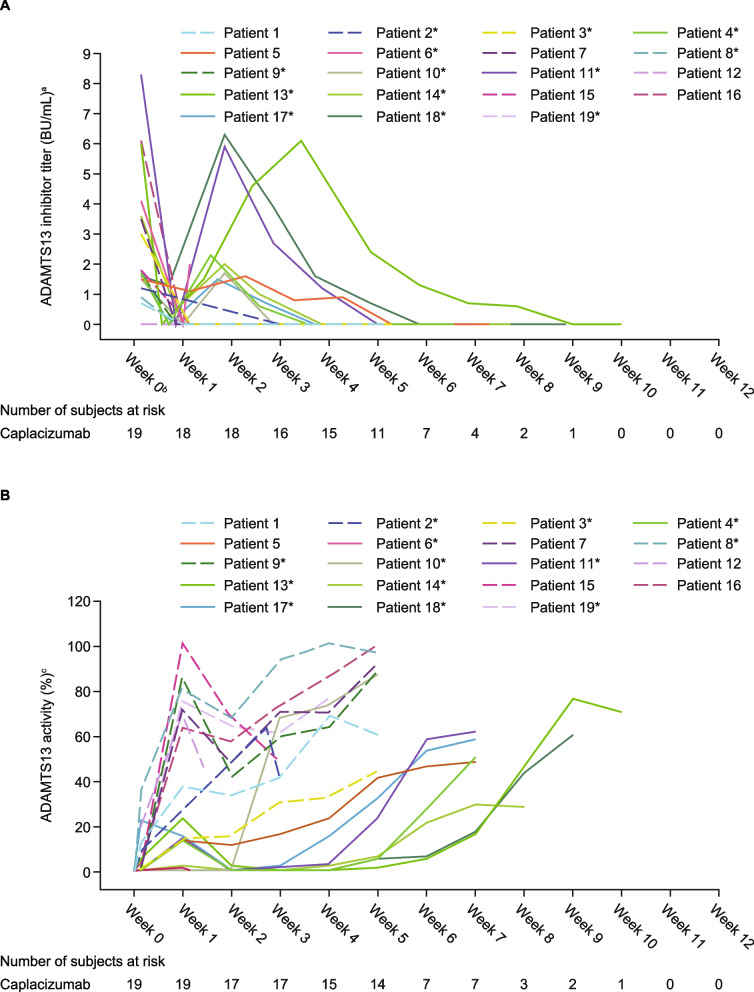
ADAMTS13 (**A**) inhibitor levels by patient (**B**) activity levels by patient. Figure 1A reprinted from *Blood* 2023*;* 142(S1): 5428; Kazunori Imada et al., Post Hoc Analysis of a Phase 2/3 Study in Japanese Patients with iTTP Treated with Caplacizumab. Copyright (2023), with permission from Elsevier. ^a^ADAMTS13 inhibitor titer data points recorded as < 0.5 were plotted as 0 for all patients. ^b^The highest value of ADAMTS13 inhibitor titer at admission, as measured by a local laboratory if available and ADAMTS13 inhibitor titer at baseline was used. ^c^ADAMTS13 activity data points < 0.5 were plotted as 0.5 for Patient 6. ADAMTS13 activity data points recorded as < 1 were plotted as 1 for Patients 3–7, 9–11, and 13–18. Solid line: Patients with ADAMTS13 inhibitor boosting. The ‘*’ symbol indicates patients who received rituximab. Patients 2, 12, 15, 19 discontinued the study before completing. ADAMTS13, a disintegrin and metalloproteinase with thrombospondin type 1 motif, member 13; mITT, modified intention-to-treat

ADAMTS13 inhibitors decreased in all patients in Week 1 after the start of treatment, but inhibitor boosting was observed in 9 patients around Week 2 (Fig. 1A). Among 9 patients with inhibitor boosting, 7 patients extended caplacizumab treatment. One patient with inhibitor boosting experienced a recurrence 2 days after discontinuing caplacizumab due to an adverse event. No other patients experienced a recurrence.

### Time to ADAMTS13 activity recovery

Median (95% confidence interval [CI]) time to recovery of ADAMTS13 activity to ≥ 10%, ≥ 20%, and ≥ 60% was 14.6 (5.9–24.8), 18.5 (5.9–31.8), and 47.5 (18.5–60.9) days, respectively (Table 2). Median time to recovery of ADAMTS13 activity was longer in patients who received rituximab versus patients who did not receive rituximab (≥ 10%: 24.8 vs 6.3 days; ≥ 20%: 31.8 vs 6.4 days; ≥ 60%: 49.8 vs 23.9 days) and in patients experiencing an initial TTP episode versus patients experiencing a subsequent TTP episode (≥ 10%: 18.5 vs 6.9 days; ≥ 20%: 28.8 vs 13.7 days; ≥ 60%: 47.5 vs 18.6 days). Patients with ADAMTS13 inhibitor levels ≥ 2 BU/mL at baseline (n = 7) took approximately twice as long to recover ADAMTS13 activity to ≥ 10%, ≥ 20%, and ≥ 60% versus patients with baseline inhibitor levels < 2 BU/mL (≥ 10%: 33.5 vs 14.1 days; ≥ 20%: 33.5 vs 16.1 days; ≥ 60%: 54.7 vs 23.2 days). Baseline severity, platelet count or time since TTP diagnosis did not appear to influence ADAMTS13 recovery (Table 2), but presence of inhibitor boosting did have an impact (discussed in inhibitor subanalysis section). Mean ADAMTS13 activity levels increased from 11.9% at baseline to 39.4% at the end of Week 1 post-TPE. Levels decreased to 28.2% at Week 2 post-TPE and then increased to a maximum of 49.5% by the end of Week 5 post-TPE for the patients without inhibitor boosting, reflecting resolution of the underlying disease (Fig. 1B). Median (range) ADAMTS13 activity level at caplacizumab treatment end was 62.0% (29.0–101.0), and no recurrence was reported during the follow-up period.

**Table 2:** Median (95% CI) time to ADAMTS13 activity ≥ 10%, ≥ 20%, and ≥ 60% overall and according to different clinical characteristics

Overall/Clinical characteristic	Number of patients	Time to sustained ADAMTS13 activity ≥ 10%, median (95% CI), days	Time to sustained ADAMTS13 activity ≥ 20%, median (95% CI), days	Time to sustained ADAMTS13 activity ≥ 60%, median (95% CI), days
Overall	19	14.6 (5.9–24.8)	18.5 (5.9–31.8)	47.5 (18.5–60.9)
Concomitant rituximab				
No rituximab used	6	6.3 (4.7–NC)	6.4 (4.7–NC)	23.9 (6.9–NC)
Rituximab used	13	24.8 (6.9–38.8)	31.8 (13.7–38.8)	49.8 (13.7–61.9)
Baseline ADAMTS13 inhibitor				
< 2 BU/mL	12	14.1 (4.5–24.8)	16.1 (4.5–31.8)	23.2 (6.9–51.8)
≥ 2 BU/mL	7	33.5 (4.7–NC)	33.5 (4.7–NC)	54.7 (18.8–NC)
Time since iTTP diagnosis				
≤ 5 days	13	18.5 (5.9–37.8)	28.8 (5.9–37.8)	37.7 (17.6–61.9)
> 5 days	6	10.3 (4.4–NC)	14.1 (4.4–NC)	39.8 (4.5–NC)
Disease severity at baseline				
Less severe	8	10.8 (4.7–46.9)	17.7 (4.7–53.9)	60.9 (6.9–NC)
Very severe	11	17.6 (4.5–24.8)	18.5 (4.5–31.8)	19.9 (13.7–49.8)
Platelet count at baseline				
< 20*10^9^/L	9	18.5 (5.9–37.8)	28.8 (5.9–37.8)	33.7 (6.9–NC)
≥ 20*10^9^/L	9	13.7 (4.5–46.9)	14.6 (4.5–53.9)	49.8 (4.5–61.9)
Inhibitor boosting present				
No	10	6.4 (4.4–13.7)	6.4 (4.4–14.6)	18.7 (4.5–27.8)
Yes	9	35.7 (6.7–46.9)	35.7 (18.5–53.9)	56.4 (18.5–NC)
Previous iTTP episode				
No	14	18.5 (5.9–37.8)	28.8 (5.9–37.8)	47.5 (18.5–60.9)
Yes	5	6.9 (4.4–NC)	13.7 (4.4–NC)	18.6 (4.5–NC)

ADAMTS13, a disintegrin and metalloproteinase with thrombospondin type 1 motif member 13; CI, confidence interval; iTTP, immune-mediated thrombotic thrombocytopenic purpura; NC, not calculated

### Inhibitor boosting subanalysis

Patients with inhibitor boosting (*n* = 9) were generally younger than those without inhibitor boosting (*n* = 10; median (range) age 50.0 [23–84] years vs 66.5 [22–86] years) and had a lower median platelet count at baseline (12.0 [8–65] × 10^9^/L vs 31.0 [9–78] × 10^9^/L). ADAMTS13 inhibitor levels at baseline and incidence of very severe/less severe disease were similar between patients with/without inhibitor boosting (Table 3). Patients with inhibitor boosting had a delayed ADAMTS13 activity response versus patients who did not have inhibitor boosting (≥ 10%: 35.7 vs 6.4 days respectively; ≥ 20%: 35.7 vs 6.4 days respectively; ≥ 60%: 56.4 vs 18.7 days respectively; Table 2 and Fig. 1B). Median (95% CI) time to platelet count response was 2.42 (0.88–3.59) days in patients with inhibitor boosting and 3.98 (1.69–not calculated) days in patients without inhibitor boosting (Table 3). The median (range) number of days of TPE was 5.0 (3–11) and 6.5 (5–20) in patients with inhibitor boosting and without inhibitor boosting, respectively. Rituximab was used in 8/9 (88.9%) patients with inhibitor boosting and 5/10 (50.0%) patients without inhibitor boosting. Of the 8 patients with inhibitor boosting who received rituximab, 7 received it after completion of TPE. The remaining patient received rituximab treatment on the day of TPE completion; this patient had discontinued caplacizumab due to an adverse event and experienced a recurrence 2 days later. Of the 5 patients without inhibitor boosting who received rituximab, 4 (80%) received it before completion of TPE and 1 (20%) received it 2 days after completion of TPE. The median first study day of rituximab use was Day 18.5 in patients with inhibitor boosting and Day 4.0 in patients without inhibitor boosting. Two (10.5%) patients experienced recurrence, ≥ 1 major thromboembolic event, or TTP-related death. Two patients in each inhibitor boosting subgroup had ≥ 1 treatment-emergent serious adverse event.

**Table 3:** Baseline characteristics and efficacy outcomes by inhibitor boosting

	**Inhibitor boosting** **(*n*= 9)**	**No inhibitor boosting** **(*n*= 10)**
Gender, n (%)		
Male	3 (33.3)	6 (60)
Female	6 (66.7)	4 (40)
Age (years), median (range)	50.0 (23–84)	66.5 (22–86)
Previous TTP episode, n (%)		
No	9 (100)	5 (50)
Yes	0	5 (50)
Disease severity at baseline, n (%)		
Very severe	6 (66.7)	5 (50)
Less severe	3 (33.3)	5 (50)
Platelet count (10^9^/L) at baseline, median (range)	12.0 (8–65)	31.0 (9–78)
Serum creatine (µmol/L) at baseline, median (range)	67.0 (64–226)	87.0 (49–162)
Lactate dehydrogenase (U/L) at baseline, median (range)	575.0 (227–1794)	452.5 (234–947)
Cardiac troponin level (µg/L) at baseline, median (range)	0.310 (0.03– 3.52)	0.055 (0.03–7.47)
ADAMTS13 inhibitor (BU/mL) at baseline, median (range)	1.80 (1.5–8.3)	1.45 (0.5–6.1)
Time to platelet count response (days), median (95% CI)	2.42 (0.88–3.59)	3.98 (1.69–NC)
Time to normalization of all 3 organ damage marker levels (days), median (95% CI)	4.48 (0.87–11.40)	3.20 (0.79–4.98)
Number of days of TPE, median (range)	5.0 (3–11)	6.5 (5–20)
Use of concomitant rituximab, n (%)	8 (88.9)	5 (50.0)
Before completion of TPE	0	4 (40.0)
Day of TPE completion	1 (11.1)^a^	0
After completion of TPE	7 (77.8)	1 (10.0)
Time to normalization of serum creatine (days), median (95% CI)	1.83 (0.49–NC)	1.92 (0.98–NC)
Time to normalization of cardiac troponin I level (days), median (95% CI)	6.31 ((1.49–NC)	3.06 (1.92–NC)
Time to normalization of lactate dehydrogenase (days), median (95% CI)	2.54 (0.87–NC)	0.98 (0.46–4.98)

*CI* Confidence interval, *iTTP* Immune-mediated thrombotic thrombocytopenic purpura, *NC* Not calculated, *TPE* Therapeutic plasma exchange, *TTP* Thrombotic thrombocytopenic purpura

^a^One patient experienced a recurrence, 2 days after discontinuing caplacizumab due to an adverse event

## Discussion

The Phase 2/3 NCT04074187 study was the first to investigate caplacizumab treatment in Japanese patients with iTTP. Our post hoc analysis of that study shows that ADAMTS13 activity and inhibitor levels were correlated with patients’ backgrounds during iTTP treatment using caplacizumab. These results suggest that early rituximab treatment during caplacizumab alongside TPE and immunosuppression treatment may reduce the risk of recurrence due to inhibitor boosting.

In this analysis, almost 50% of patients with confirmed iTTP (*n* = 9) experienced ADAMTS13 inhibitor boosting; of these, only 1 patient experienced a recurrence, 2 days after discontinuing caplacizumab due to an adverse event. Rituximab was administered to the majority of patients with inhibitor boosting, but in all such cases rituximab was administered after TPE was completed. In addition, patients with inhibitor boosting had earlier platelet recovery and fewer days of TPE compared with patients without inhibitor boosting. It is possible that TPE was completed earlier because of earlier platelet count recovery. None of the patients who received rituximab before completion of TPE experienced inhibitor boosting, suggesting that inhibitor boosting may be avoided if rituximab are administered early in the treatment period. Disease severity and ADAMTS13 inhibitor level at baseline did not appear to influence inhibitor boosting. Median time to recovery of ADAMTS13 activity to ≥ 10, ≥ 20, and ≥ 60% in this study was 14.6 (5.9–24.8), 18.5 (5.9–31.8), and 47.5 (18.5–60.9) days, respectively, which seems to be more rapid compared to previously reported real-world evidence data (ADAMTS13 activity > 30%: 31 days; ADAMTS13 ≥ 20%: 28 days) [8, 17].

Factors which may impact on ADAMTS13 activity recovery time include inhibitor titer at baseline; patients with inhibitor titer ≥ 2 BU/mL at baseline took longer to recover ADAMTS13 activity, and ADAMTS13 activity was shown to increase more slowly in patients with ADAMTS13 inhibitor boosting. Patients in the rituximab subgroup also took longer to achieve sustained ADAMTS13 activity (at any level), but it should be noted that administration of immunosuppressant agents was at the physician’s discretion. Therefore, rituximab may have only been administered after the physician noticed that ADAMTS13 recovery was slow or that the therapeutic effect was poor, as the TTP indication for rituximab in Japan is limited for relapsed/refractory disease [18]. Caplacizumab treatment may result in a rapid platelet recovery, thus delaying the administration of rituximab. Frontline use of rituximab and caplacizumab may prevent recurrence of iTTP and ADAMTS13 inhibitor boosting. Caplacizumab inhibits thrombus formation by preventing the binding of platelets to VWF [1], while rituximab acts as an immunosuppressant by targeting the CD20 protein marker on B cells, causing their depletion [19] and thus inhibiting ADAMTS13 inhibitor production [20, 21]. ISTH guidance recommends that patients with ADAMTS13 activity < 10% of normal receive rituximab as early as possible in order to target ADAMTS13 antibodies [22]. Limitations of the study include the small population size and that there was no control group; the authors anticipate that a further analysis with a larger sample size and more time points will be required in order to fully understand the impacts of caplacizumab and rituximab in this patient population.

## Conclusion

In conclusion, these results suggest that mean time to recover ADAMTS13 activity may be more rapid than previously reported, and that time to recovery of ADAMTS13 activity is influenced by factors such as rituximab use, previous iTTP history and ADAMTS13 inhibitor level at baseline. ADAMTS13 inhibitor boosting may also be associated with longer time to recover ADAMTS13 activity. Caplacizumab in conjunction with TPE and immunosuppression may reduce the risk of inhibitor boosting if rituximab is administered early in the iTTP treatment period; therefore, these results support the use of caplacizumab in the iTTP patient population.

## Data Availability

Qualified researchers may request access to patient level data and related study documents including the clinical study report, study protocol with any amendments, blank case report form, statistical analysis plan, and dataset specifications. Patient level data will be anonymized and study documents will be redacted to protect the privacy of our trial participants. Further details on Sanofi’s data sharing criteria, eligible studies, and process for requesting access can be found at: https://www.vivli.org/.

## Abbreviations

ADAMTS13: Autoantibody-mediated severe deficiency of a disintegrin and metalloproteinase with thrombospondin type 1 motif, member 13
CI: Confidence interval
iTTP: Immune-mediated thrombotic thrombocytopenic purpura
mITT: Modified intention-to-treat
TPE: Therapeutic plasma exchange
ULN: Upper limit of normal
VWF: Von Willebrand factor
